# The Role of Low CD36 Expression in the Development of Non-Small Cell Lung Cancer and Its Potential for Therapy

**DOI:** 10.3390/cancers18020217

**Published:** 2026-01-09

**Authors:** Ran Wu, Xiaohong Xu, Danju Luo, Junhua Wu, Xiaona Chang, Chenggong Ma, Bo Huang, Jun Fan, Xiu Nie

**Affiliations:** Department of Pathology, Union Hospital, Tongji Medical College, Huazhong University of Science and Technology, Wuhan 430022, China; wuran715@hust.edu.cn (R.W.); xuxiaohong@hust.edu.cn (X.X.); danjuluo86@163.com (D.L.); wujunhua_uwh@163.com (J.W.); pathologycxn@163.com (X.C.); 564352412@163.com (C.M.); huangbo1131@126.com (B.H.)

**Keywords:** non-small cell lung cancer, CD36, tumor cell proliferation, tumor microenvironment regulation, therapeutic exploration

## Abstract

Lung cancer remains one of the most prevalent and lethal malignancies worldwide. NSCLC, which constitutes approximately 85% of cases, continues to exhibit a poor prognosis despite advancements in therapeutic interventions, underscoring the urgent necessity to elucidate its molecular mechanisms and identify novel therapeutic targets. CD36, a multifunctional transmembrane glycoprotein, is integral to lipid uptake, immune recognition, inflammatory regulation, molecular adhesion, and apoptosis. Increasing evidence implicates CD36 in the progression of various cancers. In the context of lung cancer, CD36 facilitates tumorigenesis through multiple pathways, including the remodeling of tumor cell lipid metabolism, reprogramming of tumor-associated macrophages, and modulation of immune cell functions such as those of Tregs and CD8^+^ T cells. Additionally, CD36 is intricately linked with the function of cancer-associated fibroblasts and the remodeling of the tumor stromal microvasculature. This systematic review synthesizes the mechanisms by which CD36 contributes to NSCLC proliferation, migration, epithelial–mesenchymal transition, and modulation of the tumor microenvironment. Furthermore, we explore emerging therapeutic strategies that target CD36. Regulating CD36 expression effectively intervenes in the malignant behavior of NSCLC, underscoring its potential as a promising therapeutic target and prognostic marker.

## Simple Summary

Lung cancer continues to be among the most prevalent and lethal malignancies globally, with non-small cell lung cancer (NSCLC) constituting the majority of cases and presenting significant challenges in effective treatment. CD36 is integral to lipid metabolism, immune responses, and various critical biological processes, and accumulating evidence suggests a strong association with cancer progression. This review provides a comprehensive analysis of the role of CD36 in influencing lung cancer cell proliferation, metastasis, and the regulation of the tumor microenvironment, while also examining potential therapeutic strategies targeting CD36. The insights gained from these findings aim to pave the way for advancements in precision diagnosis and treatment, with the potential to enhance diagnostic and therapeutic standards and improve outcomes for patients with lung cancer.

## 1. Introduction

Lung cancer continues to be the foremost cause of cancer-related mortality worldwide. Approximately 85% of cases are classified as NSCLC, which is characterized by a dismal 5-year overall survival rate [1]. Despite significant advancements in therapeutic strategies, including targeted therapy, immunotherapy, and combination regimens, the persistently poor prognosis highlights an urgent need for innovative treatment approaches [2].

CD36 (cluster of differentiation 36), a multifunctional glycoprotein belonging to the scavenger receptor family, is widely expressed on monocytes, macrophages, endothelial cells, adipocytes, and tumor cells [3,4]. It binds to multiple ligands such as long-chain fatty acids (LCFAs), oxidized low-density lipoprotein (oxLDL), thrombospondin-1 (TSP-1), thrombospondin-2 (TSP-2), and apoptotic cells [5]. Consequently, CD36 is implicated in a range of biological processes, including lipid metabolism, immune response, inflammation, cell adhesion, angiogenesis, and apoptosis [6].

Emerging evidence suggests that CD36 plays a critical role in tumor initiation and progression; however, its expression patterns and functional implications appear to be context- and cancer-type dependent [7]. For instance, in the context of pancreatic cancer, reduced CD36 expression is associated with increased tumor volume and decreased survival rates [8]. Immunohistochemical analysis of whole tissue sections from 109 patients diagnosed with advanced high-grade serous ovarian carcinoma (HGSOC) demonstrated that CD36 expression predominantly localized to the tumor microvasculature rather than the tumor cells themselves. Notably, positive CD36 expression in the tumor vasculature was correlated with residual disease following surgical intervention, suggesting a higher tumor burden, and was significantly linked to reduced overall survival [9]. Similarly, in breast ductal carcinoma in situ (DCIS), lower levels of CD36 expression in perilesional vasculature were associated with an elevated risk of progression to invasive ductal carcinoma (IBC), indicating that the loss of CD36 may serve as a potential prognostic marker for adverse outcomes [10]. In colorectal cancer, Fang et al. reported a progressive decline in CD36 expression from adenoma to adenocarcinoma, with a marked reduction observed in adenomas undergoing malignant transformation, thus underscoring the association between low CD36 expression and poor prognosis [11].

Although several molecules, such as CD47, have been implicated in epithelial-mesenchymal transition (EMT), cancer progression, and therapy resistance, CD36 warrants particular attention in NSCLC due to its multidimensional roles in lipid metabolic reprogramming, immune modulation, and tumor microenvironment remodeling. While CD47 primarily functions as an immune checkpoint via the “don’t eat me” signal (mediated by SIRPα) to suppress macrophage phagocytosis and promote immune evasion, CD36 operates as a metabolic–immune checkpoint that integrates nutrient sensing with intracellular signaling, macrophage polarization, T cell exhaustion, and angiogenesis [12,13,14]. Ahvati et al. further emphasize that CD36 activates the PI3K/Akt and MAPK pathways to induce cancer stem-like properties and enhance metastatic potential in NSCLC. Moreover, CD36 participates in metabolic coupling between cancer-associated fibroblasts and tumor cells via exosome-mediated lipid transfer, supporting distant colonization—a mechanism less evident for CD47. In contrast to CD47, which exhibits considerable heterogeneity across NSCLC subtypes and is influenced by various inflammatory and hypoxic factors [15], CD36 shows consistent upregulation in both EGFR- and KRAS-mutant NSCLC, suggesting its broader applicability independent of specific driver mutations. Thus, focusing on CD36 offers a more integrative perspective on the metabolic–immune–stromal network in NSCLC pathogenesis and provides a promising target for combinatorial therapeutic strategies.

In recent years, the diagnostic and therapeutic potential of CD36 in the development of lung cancer has garnered increasing attention. Sun et al. demonstrated that CD36 is generally underexpressed in lung cancer tissues compared to adjacent normal tissues, which exhibit higher methylation levels [16]. Cytological and animal studies indicate that reversing CD36 expression significantly promotes tumor cell apoptosis and inhibits tumor growth. Furthermore, another study revealed that while CD36 is abundantly expressed on the stromal microvascular endothelium in normal lung tissue, its expression is markedly diminished or absent in the microvasculature of lung cancer [10]. Additionally, CD36 is intricately associated with the establishment of an immunosuppressive tumor microenvironment in lung cancer [17]. Given these observations, a deeper understanding of the molecular mechanisms through which CD36 regulates NSCLC progression could inform new therapeutic avenues. This review systematically summarizes current knowledge on the regulatory functions of CD36 in NSCLC proliferation, migration, EMT, tumor microenvironment modulation, and epigenetic modifications. Furthermore, we also discuss emerging therapeutic strategies targeting CD36, aiming to provide a consolidated theoretical foundation and novel perspectives for the precise diagnosis and treatment of lung cancer.

## 2. Molecular Mechanisms of CD36 in Lung Cancer Development

### 2.1. Relationship of CD36 to NSCLC Cell Proliferation, Migration, and EMT

**Figure 1:**
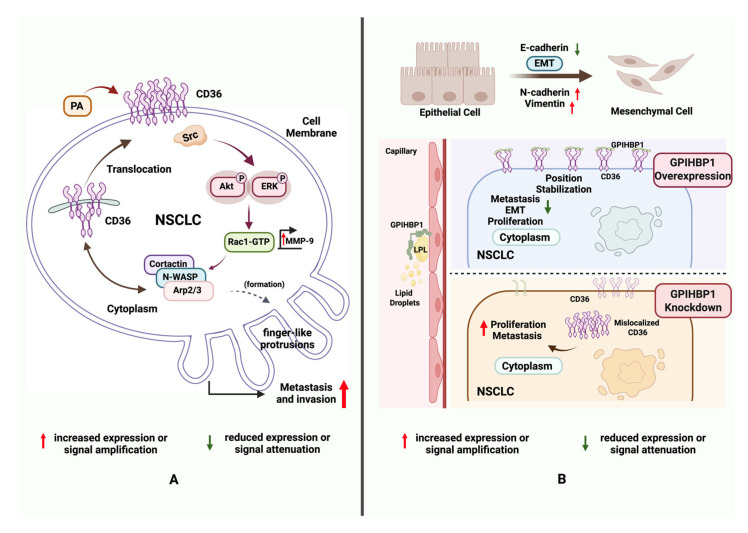
CD36 controls NSCLC metastasis, EMT, and proliferation. Figure Created in BioRender. Tooth, W. (2026) https://BioRender.com/m7wv8u0 (**A**) PA induces CD36 membrane translocation; Src-Akt/ERK activation drives Rac1/MMP-9 signaling, actin rearrangement, and protrusions, boosting NSCLC invasion. (**B**) GPIHBP1 binds CD36: overexpression stabilizes CD36 (inhibits proliferation and EMT); knockdown mislocalized CD36 (promotes metastasis). Abbreviations: NSCLC, non-small cell lung cancer; PA, palmitic acid; MMP-9, matrix metallopeptidase 9; GPIHBP1, glycosylphosphatidylinositol-anchored high-density lipoprotein-binding protein 1.

Research on lung adenocarcinoma (LUAD) has demonstrated that palmitic acid (PA) and a high-fat diet (HFD) promote LUAD cell proliferation in a CD36-dependent manner [18]. In lung cancer cell models, PA stimulation induces the translocation of CD36 from the cytoplasm to the cell membrane, where it interacts with Src kinase. This interaction activates downstream Akt and ERK signaling pathways, thereby facilitating LUAD cell proliferation and metastasis. Notably, the study also found that PA treatment enhances CD36 sarcolemmal translocation via the Src-Akt/ERK pathway, activates Rac1, upregulates matrix metallopeptidase 9 (MMP-9), and causes the redistribution of actin assembly proteins such as cortactin, N-WASP, and Arp2/3. These discrete structures play a critical role in mediating cell migration and invasion [19]. Ultimately, this process results in the development of finger-like protrusions that enhance the migratory capacity of cells. In vivo experiments have further demonstrated that the knockdown of CD36 significantly inhibits the growth and lung metastasis of LUAD xenograft tumors in mice subjected to a high-fat diet. These findings elucidate the mechanisms by which CD36 facilitates lung cancer cell invasion and metastasis through the regulation of cytoskeletal remodeling and signaling pathways (Figure 1A). Additionally, a separate study on non-small cell lung cancer (NSCLC) observed that the upregulation of miR-21 enhances NSCLC cell migration via CD36-mediated fatty acid metabolism, while the inhibition of CD36 suppresses this migration, thereby reinforcing the pivotal role of CD36 in lung cancer cell motility [20].

EMT is a key process through which tumor cells acquire migratory and invasive capabilities, facilitating metastasis [21]. It plays a significant role in the progression of lung cancer [22]. Research indicates that CD36 plays a significant role in promoting the EMT in non-small cell lung cancer (NSCLC) cells. Glycosylphosphatidylinositol-anchored high-density lipoprotein-binding protein 1 (GPIHBP1), a crucial protein involved in lipid metabolism predominantly expressed in capillary endothelial cells, facilitates the lipolytic processing of triglyceride-rich lipoproteins by binding and transporting lipoprotein lipase to the capillary lumen [23]. Overexpression of GPIHBP1 has been shown to inhibit NSCLC cell migration, proliferation, and the EMT process, while silencing GPIHBP1 produces the opposite effects (Figure 1B). The underlying mechanism involves a direct interaction between GPIHBP1 and CD36. Silencing GPIHBP1 disrupts the localization of CD36, thereby promoting tumor progression and metastasis through sarcolemmal translocation [24]. This suggests that abnormal localization or function of CD36 may directly drive EMT, endowing lung cancer cells with more aggressive biological behaviors.

Furthermore, Chen et al. demonstrated that the long non-coding RNA (lncRNA) CCAT1 facilitates the nuclear translocation of fatty acid-binding protein 5 (FABP5), thereby promoting the formation of the Retinoid X receptor (RXR)/PPAR-γ transcriptional complex [25]. This complex subsequently activates the transcription of CD36, Pyruvate dehydrogenase kinase 1 (PDK1), and VEGFA, leading to an acceleration of LUAD cell proliferation and angiogenesis. This finding suggests that CD36 may also indirectly affect lung cancer cell proliferation, migration, and EMT through its interactions with other molecules or signaling pathways.

### 2.2. Regulatory Role of CD36 in the NSCLC Tumor Microenvironment

In addition to its direct effects on tumor cells, CD36 modulates the tumor microenvironment through various mechanisms. The tumor microenvironment (TME) is a critical determinant of tumor development, progression, and metastasis [26]. CD36 exerts complex and diverse regulatory functions within multiple components of the TME, including immune cell function, angiogenesis, and interactions with other stromal cells [27].

#### 2.2.1. CD36 Regulates the Polarization State of Tumor-Associated Macrophages (TAMs)

**Figure 2:**
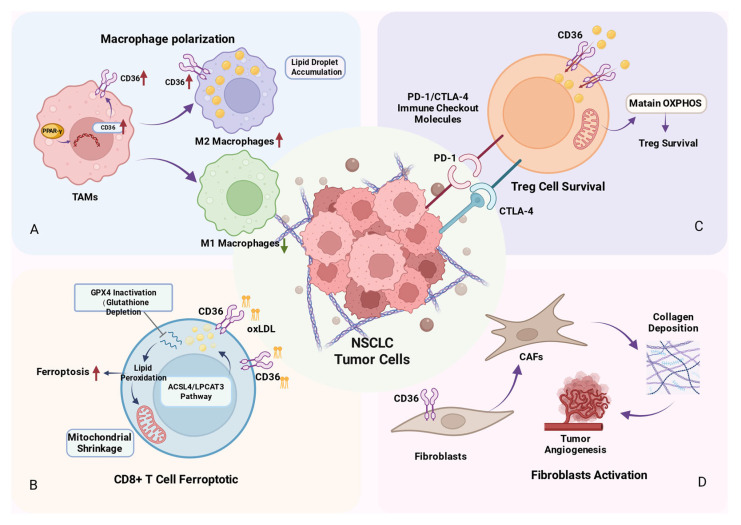
Schematic of CD36-mediated mechanisms within the immune microenvironment of NSCLC. Figure created in BioRender. Tooth, W. (2025) https://BioRender.com/aubwhre (**A**) CD36 promotes the polarization of TAMs towards the M2 phenotype (characterized by lipid droplet accumulation) and suppresses the M1 phenotype. (**B**) CD36 induces ferroptosis in CD8^+^ T cells via enhancing lipid peroxidation. (**C**) CD36 sustains the survival of regulatory Tregs by maintaining oxidative phosphorylation (OXPHOS). (**D**) CD36 drives the activation of fibroblasts into cancer-associated fibroblasts (CAFs), which support angiogenesis and collagen deposition. Abbreviations: ACSL4, acyl-CoA synthetase long-chain family member 4; LPCAT3, Lyso-PL Acyltransferases 3; OXPHOS, oxidative phosphorylation; PD-1, programmed cell death protein 1; CTLA-4, cytotoxic T-lymphocyte-associated protein 4; CAFs, cancer-associated fibroblasts.

Macrophages represent one of the most prevalent immune cell types within the TME, and their polarization state has a direct impact on tumor progression [28]. TAMs constitute a heterogeneous group of myeloid cells that play a critical role in shaping the inflammatory profile of the TME [29]. Contemporary research is concentrated on the phenotypic characterization of M1 and M2 macrophages, with M1 macrophages generally recognized for their pro-inflammatory properties, whereas M2 macrophages are typically associated with tumor progression [30]. As a key surface molecule on macrophages, CD36 expression and function have become a research hotspot. In studies concerning lung cancer, a traditional Chinese medicinal formula, Wen Xia Chang Fu Formula (WCF), has been demonstrated to reprogram TAMs via the peroxisome proliferator-activated receptor γ (PPAR-γ)/CD36 signaling pathway, thereby inhibiting metastasis in Lewis lung carcinoma (LLC) models [31]. Treatment with WCF significantly downregulated markers associated with M2-like polarization while upregulating markers indicative of M1-like polarization in macrophages (Figure 2A). This shift in polarization directly attenuated the migratory and invasive capabilities of LLC cells. In vivo experiments corroborated these findings, showing a substantial reduction in the bioluminescent signal from LLC-Luc cells following WCF treatment. This effect was contingent upon the regulation of fatty acid metabolism, achieved by decreasing lipid accumulation in M2 macrophages and inhibiting the activity of the PPAR-γ/CD36 pathway, ultimately leading to the reprogramming of TAM polarization.

Additionally, Ni et al. found that deletion of tumor necrosis factor receptor-associated factor 3 (TRAF3) increased expression of M1 markers, inducible nitric oxide synthase (iNOS), FGR, SLC4A7, and decreased expression of M2 markers, CD206, CD36, and ABCC3 in macrophages [32]. These findings indicate that CD36 plays a significant role in the regulation of macrophage polarization.

#### 2.2.2. CD36 Regulates Lipid Metabolism and Ferroptosis in CD8^+^ T Cells

CD8^+^ T cells, as primary cytotoxic T lymphocytes, are integral to immune defense and anti-tumor immunity due to their ability to directly eliminate target cells [33]. CD36 facilitates the uptake of fatty acids by tumor-infiltrating CD8^+^ T cells [34]. An accumulation of intracellular fatty acids can induce lipid peroxidation, thereby activating the ferroptosis pathway [35,36]. Ferroptosis directly impairs CD8^+^ T cell function [37]. It significantly reduces the production of cytotoxic cytokines and diminishes their specific killing capacity against tumor cells, ultimately weakening anti-tumor activity and reinforcing the immunosuppressive state of the TME (Figure 2B). Functional validation experiments further revealed that tumor growth rates were markedly lower in CD36-deficient melanoma mouse models. CD36 deficiency has been shown to protect CD8^+^ tumor-infiltrating lymphocytes from dysfunction induced by lipid peroxidation and ferroptosis, thereby preserving their anti-tumor capacity [38]. Similar mechanisms have been observed in lung cancer research. 18β-Glycyrrhetinic acid (GA) was shown to inhibit CD36 expression, reduce arachidonic acid (AA)-mediated ferroptosis in CD8^+^ T cells, and consequently prevent their functional exhaustion. This process enhances the body’s anti-tumor immune response and effectively inhibits the growth of Lewis lung cancer [39]. Collectively, these studies collectively demonstrate that within the TME, CD36 can regulate lipid metabolism and ferroptosis in CD8^+^ T cells, leading to their dysfunction and contributing to an immunosuppressive state.

In NSCLC tumor tissues, the accumulation of CD36^+^CD8^+^T cells correlates with advanced clinical stage, larger tumor size, and lymph node metastasis. Moreover, high infiltration of CD36^+^CD8^+^T cells indicates poorer prognosis in terms of overall survival (OS) and recurrence-free survival (RFS), and a poorer response to chemotherapy. These CD36^+^CD8^+^T cells exhibit reduced levels of GZMB and IFN-γ, and elevated levels of PD-1 and TIGIT. Analysis of the tumor-infiltrating immune landscape revealed that CD36^+^CD8^+^T cells exhibit a positive correlation with regulatory T cells (Tregs) and M2 macrophages, while displaying a negative correlation with Th1 cells. Importantly, inhibition of CD36 has been shown to partially restore the cytotoxic function of CD8^+^ T cells, resulting in enhanced production of GZMB and IFN-γ. These findings indicate that CD36^+^CD8^+^T cells possess impaired immune functionality, and their elevated infiltration is associated with poor prognosis and suboptimal chemotherapy response in patients with NSCLC. Consequently, CD36 emerges as a potential therapeutic target when used in conjunction with chemotherapy for NSCLC [40].

Furthermore, accumulated cholesterol in the TME can induce increased CD36 expression in tumor-infiltrating CD8^+^ T cells, a change associated with malignant tumor progression and poor patient prognosis [41]. These studies suggest that CD36 may play a key role in regulating tumor immune evasion mediated by tumor-infiltrating CD8^+^ T cells and the efficacy of immunotherapy.

#### 2.2.3. Role of CD36 in Regulating Metabolic Adaptation of Treg Cells

Treg cells, as immunosuppressive cells, are a major contributor to therapeutic resistance in tumors [42]. Wang et al. found high CD36 expression in Treg cells within clinical samples and mouse lung cancer models [43]. Treg cells with low CD36 expression exhibited a reduction in mitochondrial number, decreased cristae density, and a significantly diminished mitochondrial membrane potential, thereby impairing their oxidative phosphorylation (OXPHOS) capability. Administration of a PPAR-β agonist mitigated the effects of CD36 deficiency, restoring mitochondrial function and reducing apoptosis in intratumoral Treg cells within the lung cancer model. These findings indicate that the CD36-PPAR-β axis constitutes a fundamental pathway for the metabolic adaptation of Treg cells in the context of lung cancer. The elevated lactate environment characteristic of the lung cancer TME can adversely affect immune cell survival [44]. By sustaining mitochondrial OXPHOS, CD36 enhances the NAD/NADH ratio in intratumoral Treg cells. NAD, a critical coenzyme in lactate metabolism, facilitates the conversion of lactate to pyruvate for energy production, thereby enabling Treg cells to withstand lactate-induced stress within the lung cancer TME (Figure 2C). This study suggests that CD36 serves a protective function in lung cancer Treg cells, preserving their immunosuppressive activity. Additionally, the study found that specific knockout of CD36 in Treg cells in melanoma and colon cancer models reduced intratumoral Treg cell numbers while increasing CD8^+^ T cell numbers, thereby delaying tumor progression. These studies indicate that CD36 positively regulates Treg cell survival within the TME, playing a significant role in maintaining an immunosuppressive microenvironment.

#### 2.2.4. Role of CD36 in Cancer-Associated Fibroblasts (CAFs)

Cancer-associated fibroblasts (CAFs) are pivotal components of the TME [45]. CAFs constitute the primary matrix components [46], promoting tumor growth and metastasis by secreting factors that drive tumorigenesis and stimulating angiogenesis and cancer cell proliferation (Figure 2D).

Li et al. co-cultured NIH-3T3 mouse embryonic fibroblasts with EMT mouse breast cancer cells and stained them with labeled antibodies for α-SMA and CD36 at different time points [47]. Initially, α-SMA expression was negative, while CD36 was highly expressed on the cell membrane of normal fibroblasts. As co-culture time increased, α-SMA expression increased in CAFs. Nevertheless, CD36 exhibited an inverse pattern, progressively diminishing as fibroblasts transitioned into CAFs. This suggests that CD36 plays a significant role in the CAF formation process.

A separate study employed RNA sequencing to identify specific markers distinguishing CAFs from normal fibroblasts (NFs) in NSCLC. The findings revealed that CD36 expression was elevated in CAFs compared to NFs. Furthermore, when comparing lung tissue-derived CAFs (Lung-CAFs) and NFs, CD36 levels were even more pronounced in lymph node-derived CAFs (LN-CAFs), potentially contributing to the maintenance of a lipid metabolism-driven tumor survival state in metastatic lymph nodes [48]. This indicates that CD36 expression levels in CAFs may be associated with aggressive biological behavior.

### 2.3. Regulatory Role of CD36 in Tumor Angiogenesis

CD36 was initially identified as an anti-angiogenic receptor for TSP-1 [49]. TSP-1 and TSP-2, as endogenous angiogenesis inhibitors, can directly regulate endothelial cell migration, proliferation, survival, and apoptosis through molecules like CD36, CD47, and integrins, and antagonize the pro-angiogenic activity of vascular endothelial growth factor (VEGF) [50,51]. CD36 collaborates with β1 integrin to facilitate the transmission of signals initiated by TSP-1 and TSP-2, and it can also associate with vascular endothelial growth factor receptor 2 (VEGFR2), thereby forming a platform that integrates pro- and anti-angiogenic signals [52]. Subsequent research has demonstrated that TSP-1, through CD36, inhibits the uptake of myristic acid by endothelial cells, which in turn impedes nitric oxide (NO) signaling and ultimately suppresses angiogenesis [53]. Additional experiments have confirmed that systemic administration of TSP-1 peptide mimetics induces apoptosis in endothelial cells via CD36, significantly inhibiting melanoma metastasis and the growth and vascularization of human pancreatic cancer xenografts. This underscores the pivotal anti-angiogenic function of CD36 as a receptor for TSP-1 [54]. Moreover, studies suggest that CD36 is involved in the regulation of signaling pathways such as PI3K/AKT/mTOR, influencing EMT and extracellular matrix (ECM) remodeling by modulating downstream molecules like E-cadherin and vimentin, thereby facilitating the formation of vasculogenic mimicry (VM). Additionally, CD36, upon binding to THBS2, can indirectly regulate VM by recruiting SHP-1 and impacting the VEGF-A/VEGFR2 pathway [55,56]. CD36 has been identified as a mediator of tumor cell adhesion to laminin within the extracellular matrix (ECM), thereby providing a structural foundation for the formation of vasculogenic mimicry (VM) tubular structures and facilitating tumor angiogenesis. This evidence underscores the critical role of CD36 in both physiological and tumor-related angiogenesis.

**Figure 3:**
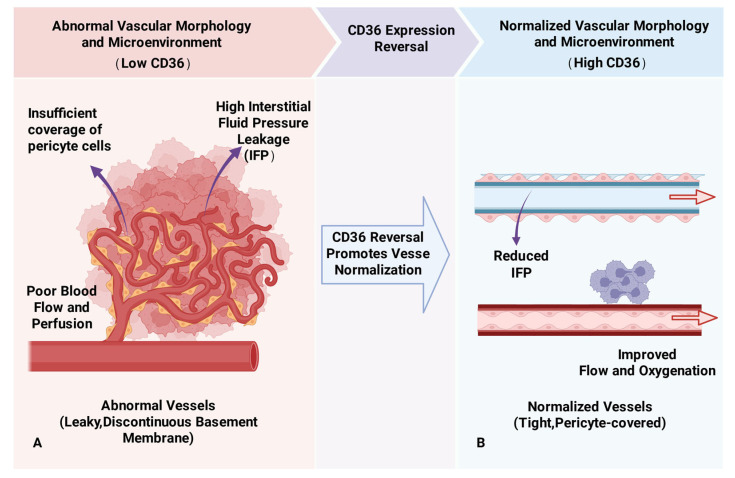
Schematic of CD36 reversal-driven tumor vessel normalization. Figure created in BioRender. Tooth, W. (2025) https://BioRender.com/zq1tptz: (**A**) Under conditions of low CD36 expression, tumor vessels exhibit an abnormal morphology: they are leaky with a discontinuous basement membrane, have insufficient pericyte coverage, and are characterized by poor perfusion and high interstitial fluid pressure (IFP). (**B**) Restoration of CD36 expression promotes vessel normalization, leading to tight, pericyte-covered vessels, along with reduced IFP and improved blood flow and oxygenation. Abbreviations: IFP, interstitial fluid pressure.

Currently, the relationship between CD36 and angiogenesis in NSCLC has limited exploratory research. Hale et al. observed increased vascular density and larger tumor volumes in CD36 knockout mice compared to wild-type mice in a Lewis lung cancer model, suggesting CD36 may have anti-angiogenic functions in NSCLC [57]. Cai et al., through public database analysis and immunohistochemical validation, found low CD36 expression in lung cancer tissues, predominantly localized to microvessels in the alveolar walls [10]. Tumor vasculature, in contrast to normal vasculature, often displays morphological and functional abnormalities, such as inadequate pericyte coverage, excessive dilation, and discontinuous basement membranes. These characteristics contribute to vascular leakage, elevated interstitial osmotic pressure, and diminished blood flow and perfusion levels [58]. Reduced blood flow not only impedes the infiltration of anti-tumor immune cells but also hinders the delivery of chemotherapy agents and immune cells to the tumor site (Figure 3A), thereby facilitating immune escape [59]. In recent years, strategies targeting tumor vasculature have shifted from anti-angiogenesis to normalization of vascular structure and function [60]. Therefore, restoring CD36 expression in lung cancer may promote tumor vessel normalization, improve the TME, and inhibit lung cancer growth, invasion, metastasis, and disease progression risk (Figure 3B).

## 3. CD36-Based Therapeutic Strategies and Progress in Lung Cancer

CD36 alters the metabolic profile of lung cancer cells by reprogramming their lipid metabolism, circumventing drug-induced inhibition of glucose metabolism. Simultaneously, it scavenges reactive oxygen species through fatty acid metabolism, thereby reducing apoptosis sensitivity and conferring drug resistance [61]. Concurrently, CD36 modulates the tumor immune microenvironment by facilitating the adaptation of Tregs to high-lactate tumor microenvironments and promoting ferroptosis in CD8^+^ T cells, thereby weakening anti-tumor immune responses and exacerbating resistance to lung cancer immunotherapy. Additionally, drug efflux driven by high osmotic pressure in tumor vasculature contributes to the development of drug resistance. Currently, therapeutic strategies targeting CD36 are under active investigation, encompassing natural medicines, existing pharmaceuticals, drug combinations, and agents related to epigenetic regulation.

### 3.1. Exploration of Drugs Regulating CD36 Expression

Natural products have attracted considerable attention due to their multi-target effects and relatively low side effect profiles, with some demonstrating anti-lung cancer effects through the modulation of CD36. Research indicates that the traditional Chinese medicine WCF modulates TAMs through the PPAR-γ/CD36 signaling pathway, thereby inhibiting the metastasis of Lewis lung cancer. This implies that WCF exerts its anti-metastatic effects by regulating CD36-mediated lipid metabolism and macrophage polarization [31]. Furthermore, another study has demonstrated that 18β-Glycyrrhetinic acid, a principal metabolite of glycyrrhizic acid, inhibits AA-induced ferroptosis in CD8^+^ T cells by downregulating CD36 expression. This action enhances anti-tumor immune responses and suppresses the growth of LLC [39]. These findings elucidate a novel mechanism underlying GA’s anti-cancer and immunomodulatory properties and underscore its potential as an immunopotentiator in lung cancer therapy.

Several existing pharmacological agents and their combinations have been identified as modulators of CD36 in the context of lung cancer treatment. Research indicates that aspirin inhibits the progression of NSCLC by precisely regulating the localization of CD36, a process mediated by GPIHBP1 [24]. Notably, GPIHBP1 expression is diminished in lung cancer tissues, and aspirin administration has been shown to enhance its levels. Overexpression of GPIHBP1 results in the inhibition of NSCLC cell migration, proliferation, and EMT, whereas silencing GPIHBP1 yields the opposite effects. Given that GPIHBP1 directly interacts with CD36, its knockdown disrupts the localization of CD36, thereby facilitating tumor progression and metastasis. In breast cancer, combination strategies involving CD36-targeted drugs and targeted therapies demonstrate significant efficacy. For HER2-resistant PTEN-deficient breast cancer cells, CD36 inhibitors synergistically enhance the antiproliferative effects of PI3K inhibitors (such as alpelisib and inavolisib) by simultaneously targeting the PI3K signaling pathway and exogenous fatty acid uptake to overcome drug resistance [62].

**Figure 4:**
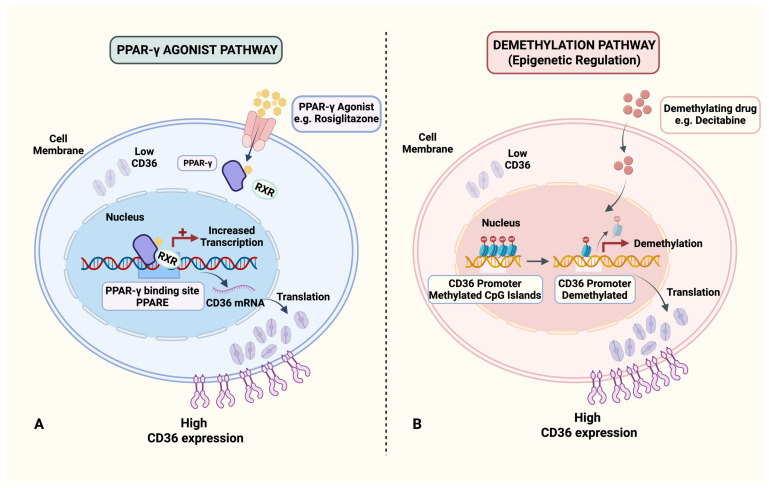
Two pathways for reversing CD36 expression via pharmacological interventions. Figure created in BioRender. Tooth, W. (2026) https://BioRender.com/blyrspo (**A**) PPAR-γ agonist (e.g., rosiglitazone) promotes PPAR-γ/RXR binding to the CD36 promoter (PPARE), enhancing CD36 transcription and expression. (**B**) Demethylating drug (e.g., decitabine) induces demethylation of methylated CpG islands in the CD36 promoter, thereby increasing CD36 expression. Abbreviations: PPAR-γ, peroxisome proliferator-activated receptor gamma; RXR, retinoid X receptor; PPARE, PPAR response element.

The CD36 gene promoter region contains a specific PPAR-γ response element. Upon ligand activation, PPAR-γ binds to this element, forming a transcriptional complex that directly initiates CD36 gene transcription and promotes CD36 protein synthesis [63]. Studies indicate that PPAR-γ agonists like troglitazone and rosiglitazone can significantly increase CD36 expression in endothelial cells, restoring their sensitivity to TSP-1 [64]. In various cell types within the tumor stroma, CD36 expression is extremely low compared to surrounding disease-free tissue. McCarty et al. found that PPAR-γ agonists improve tumor vasculature by upregulating CD36 expression, thereby enhancing the efficacy of paced chemotherapy [65]. Caruso et al. found that in invasive breast ductal samples, CD36 and PPAR-γ were prominently expressed in the intralobular capillaries of peritumoral disease-free tissue but were absent in tumor stromal capillary endothelial cells (ECs) and pericytes [10]. In cases of DCIS, lower CD36 expression in tumor stromal capillaries correlated with a higher likelihood of progression to invasive breast cancer. In mouse xenograft models, early intervention with the PPAR-γ agonist rosiglitazone significantly reduced tumor volume/weight, vascularization, and cell proliferation, showing notable anti-tumor effects. In research related to NSCLC, Keshamouni et al. found PPAR-γ activation inhibits tumor-associated angiogenesis by blocking the expression of angiogenesis-related chemokines [66]. PPAR-γ agonists may impede lung cancer growth through remodeling of the tumor microvasculature, although further validation is required (Figure 4A). Furthermore, in ovarian cancer research, Ning et al. discovered that the PARP inhibitor (PARPi) niraparib enhances CD36 expression and induces fatty acid accumulation and ferroptosis in ovarian cancer cells in an NRF2-dependent manner, significantly inhibiting metastatic lesions [67]. The potential role of PARP in exerting anti-tumor effects in NSCLC through the modulation of CD36 expression warrants further investigation.

### 3.2. Exploration of Epigenetic Regulation-Related Drugs

Sun et al. utilized microarray analysis, qRT-PCR, and Western blotting to confirm the low expression and hypermethylation of CD36 in lung cancer tissues [16]. Subsequent research demonstrated that a combined treatment with decitabine and chidamide could reverse the methylation of CD36, upregulate its expression, and synergistically inhibit tumor growth. This finding suggests that strategies aimed at restoring CD36 expression through demethylation may represent a promising therapeutic approach for lung cancer (Figure 4B). However, it is important to note that the demethylating agent decitabine employed in this study is not specific to CD36, raising a critical question regarding whether its tumor-suppressive effects may be influenced by interactions with other molecules or pathways in vivo, which necessitates further exploration. In related research on colorectal cancer (CRC), investigators have identified abnormal hypermethylation of the zinc finger protein ZNF334 in CRC tissues compared to normal tissues. Building on this observation, researchers developed a DNA demethylating agent specifically targeting the ZNF334 promoter, successfully restoring its expression. Subsequent in vitro and in vivo experiments have confirmed that this targeted demethylating drug effectively upregulates ZNF334 expression and significantly inhibits CRC growth [68]. This finding suggests that the design of specific targeted demethylating drugs based on the CD36 sequence may inhibit tumor progression, thereby offering a novel research direction for optimizing precise therapeutic strategies in lung cancer.

### 3.3. CD36-Based Targeted Therapy and Application of Immune Checkpoint Inhibitors

Molecular targeted therapy is a treatment approach that intervenes on tumor-specific molecular targets (such as receptors, key enzymes in signaling pathways, etc.) [69]. The expression differences of CD36 in various malignant tumors and its relationship with prognosis, clinical stage, and immune invasion make it promising to become a biomarker for tumor prognosis assessment [70]. Recent studies have shown that CD36 is a key molecule that regulates the response of tumors to existing targeted and immunotherapies. CD36 mediates drug endocytosis as a membrane receptor, facilitating intracellular drug delivery through the EEA1/Rab5 endocytosis cascade. Clinical studies on PROTAC drugs reveal that knocking down CD36 reduces tumor cell uptake by 19.6-fold, resulting in nearly complete loss of tumor suppression activity [71]. This characteristic of CD36 offers novel research avenues for tumor-targeted therapies. In pancreatic cancer, Obacunone enhances the anti-tumor effect of PD-1 inhibitors by regulating CD36 [72]. In osteosarcoma, targeting oxLDL-mediated reprogramming of CD36^+^ CAF can significantly enhance the efficacy of PD-1 immunotherapy [73]. In gastric cancer, the CD36-BATF2/MYB signaling axis has been proven to predict the therapeutic response of patients to PD-1 inhibitors [74]. These studies collectively suggest that CD36 not only participates in tumor metabolism and microenvironment remodeling but may also become an important synergistic target for combined immune checkpoint inhibitors, providing a new direction for overcoming immunotherapy resistance.

## 4. Conclusions

Lung cancer continues to exhibit the highest global incidence and mortality rates among malignancies, representing a significant and ongoing threat to human health. The pathogenesis of lung cancer, particularly NSCLC, remains only partially understood, prompting the pursuit of novel molecular targets and therapeutic approaches. CD36, a multifunctional scavenger receptor class B transmembrane glycoprotein, has emerged as a pivotal factor in both physiological processes and cancer biology. It plays a crucial role in lipid uptake and metabolism, immune recognition, inflammatory regulation, cell adhesion, and apoptosis. Recently, the role of CD36 in lung cancer development has garnered increasing attention due to its multifaceted involvement in tumor initiation, progression, and resistance to therapy.

In NSCLC, CD36 demonstrates context-dependent roles. Evidence from cellular and animal studies suggests that CD36 functions as an oncogenic driver by enhancing tumor cell proliferation, migration, invasion, and EMT. These effects are mediated through mechanisms such as facilitating fatty acid uptake, activating Src/Akt/ERK signaling pathways, and inducing cytoskeletal remodeling. Furthermore, CD36 plays a significant role in reprogramming the TME. It modulates the polarization of TAMs towards a pro-tumorigenic M2 phenotype via the PPAR-γ/CD36 axis, promotes ferroptosis and impairs CD8^+^ T cell function through lipid peroxidation, and supports the survival and immunosuppressive function of regulatory Tregs by maintaining their oxidative phosphorylation metabolism. Additionally, CD36 affects CAF activation and regulates tumor angiogenesis, where its deficiency leads to abnormal, leaky vasculature that hinders drug delivery and immune cell infiltration.

Notably, CD36 expression is frequently downregulated in NSCLC tissues compared to adjacent normal lung, and this reduction is often associated with promoter hypermethylation. Its expression pattern is also spatially distinct, being predominantly localized to the tumor-associated vasculature rather than cancer cells themselves in many cases. Restoring CD36 expression presents a promising therapeutic avenue, as it may normalize tumor vasculature, reverse CAF activation, and alleviate immunosuppression. Several therapeutic strategies targeting CD36 or its regulatory pathways show translational potential. These include natural compounds (e.g., Wenxia Changfu Formula, 18β-Glycyrrhetinic acid), repurposed drugs (e.g., aspirin), PPAR-γ agonists (e.g., rosiglitazone), and epigenetic modifiers (e.g., decitabine). Preclinical evidence suggests that PPAR-γ agonists can upregulate CD36 transcription, improve vascular structure, and synergize with immune checkpoint inhibitors like PD-1 blockers. Similarly, demethylating agents may reverse CD36 silencing and exert anti-tumor effects. Furthermore, CD36 itself is being explored as a biomarker for prognosis and response prediction, as well as a potential target for drug-delivery systems given its role in endocytosis.

Despite recent advancements, research on CD36 in NSCLC remains in a developmental stage. Critical challenges and future research directions involve elucidating the precise and potentially contradictory roles of CD36 across various cellular compartments, including tumor cells, vasculature, and immune cells. Additionally, it is essential to understand the determinants of its context-dependent expression and function and to develop more specific and potent CD36-targeted agents. The integration of CD36-directed therapies with existing treatment modalities—such as chemotherapy, targeted therapy, and immunotherapy—represents a promising avenue for overcoming treatment resistance. In summary, CD36 occupies a pivotal position at the intersection of metabolism, immunity, and stroma in NSCLC, presenting a unique and integrative target with significant potential to enhance the precision diagnosis and treatment of this debilitating disease.

## Data Availability

No new data were created or analyzed in this study. Data sharing is not applicable to this article.

## Abbreviations

The following abbreviations are used in this manuscript:

NSCLC	Non-small cell lung cancer
CD36	Directory of Open Access Journals
LCFAs	Long-chain fatty acids
ox LDL	Oxidized low-density lipoprotein
TSP	Thrombospondin
HGSOC	High-grade serous ovarian carcinoma
DCIS	Ductal carcinoma in situ
IBC	Invasive ductal carcinoma
EMT	Epithelial–mesenchymal transition
LUAD	Lung adenocarcinoma
PA	Palmitic acid
HFD	High-fat diet
MMP-9	Matrix metallopeptidase 9
GPIHBP1	Glycosylphosphatidylinositol-anchored high-density lipoprotein-binding protein 1
lncRNA	Long non-coding RNA
CCAT1	Colon cancer-associated transcript 1
FABP5	Fatty acid-binding protein 5
RXR	Retinoid X receptor
PPAR-γ	Peroxisome proliferator-activated receptor γ
PDK1	Pyruvate dehydrogenase kinase 1
VEGFA	Vascular endothelial growth factor A
TME	Tumor microenvironment
CAFs	Cancer-associated fibroblasts
TAMs	Tumor-associated macrophages
ACSL4	Acyl-CoA synthetase long-chain family member 4
LPCAT3	Lysophosphatidylcholine acyltransferase 3
OXPHOS	Oxidative phosphorylation
CTLA-4	Cytotoxic T-lymphocyte-associated protein 4
LLC	Lewis lung carcinoma
TRAF3	Tumor necrosis factor receptor-associated factor 3
iNOS	Inducible nitric oxide synthase
ABCC3	ATP-binding cassette subfamily C member 3
GA	Glycyrrhetinic acid
AA	Arachidonic acid
OS	Overall survival
RFS	Recurrence-free survival
NAD	Nicotinamide adenine dinucleotide
7NFs	Normal fibroblasts
Lung-CAFs	Lung tissue-derived cancer-associated fibroblasts
LN-CAFs	Lymph node-derived cancer-associated fibroblasts
VEGF	Vascular endothelial growth factor
VEGFR	vascular endothelial growth factor receptor
PI3K	Phosphatidylinositol 3-kinase
ECM	Extracellular matrix
VM	Vasculogenic mimicry
IFP	Interstitial fluid pressure
NRF2	Nuclear factor erythroid 2-related factor 2
CRC	Colorectal cancer

## References

[1] Thai A.A., Solomon B.J., Sequist L.V., Gainor J.F., Heist R.S. (2021). Lung cancer. Lancet.

[2] Xie S., Wu Z., Qi Y., Wu B., Zhu X. (2021). The metastasizing mechanisms of lung cancer: Recent advances and therapeutic challenges. Biomed. Pharmacother..

[3] Yue H., Febbraio M., Klenotic P.A., Kennedy D.J., Wu Y., Chen S., Gohara A.F., Li O., Belcher A., Kuang B. (2019). CD36 Enhances Vascular Smooth Muscle Cell Proliferation and Development of Neointimal Hyperplasia. Arter. Thromb. Vasc. Biol..

[4] Płóciennikowska A., Hromada-Judycka A., Borzęcka K., Kwiatkowska K. (2015). Co-operation of TLR4 and raft proteins in LPS-induced pro-inflammatory signaling. Cell Mol. Life Sci..

[5] Proulx C., Zhang J., Sabatino D., Chemtob S., Ong H., Lubell W.D. (2020). Synthesis and Biomedical Potential of Azapeptide Modulators of the Cluster of Differentiation 36 Receptor (CD36). Biomedicines.

[6] Xia L., Zhou Z., Chen X., Luo W., Ding L., Xie H., Zhuang W., Ni K., Li G. (2023). Ligand-dependent CD36 functions in cancer progression, metastasis, immune response, and drug resistance. Biomed. Pharmacother..

[7] Chen Y.J., Liao W.X., Huang S.Z., Yu Y.F., Wen J.Y., Chen J., Lin D.G., Wu X.Y., Jiang N., Li X. (2021). Prognostic and immunological role of CD36: A pan-cancer analysis. J. Cancer.

[8] Jia S., Zhou L., Shen T., Zhou S., Ding G., Cao L. (2018). Down-expression of CD36 in pancreatic adenocarcinoma and its correlation with clinicopathological features and prognosis. J. Cancer.

[9] Grech C.T., Aust S., Hofstetter G., Uhl E., Hinterleitner L., Grimm C., Polterauer S., Pils D. (2025). High CD36 expression in the tumor microenvironmental vasculature correlates with unfavorable overall survival in high grade serous ovarian cancer. Sci. Rep..

[10] Caruso J.A., Wang X., Murrow L.M., Rodriguez C.I., Chen-Tanyolac C., Vu L., Chen Y.Y., Gascard P., Gartner Z.J., Kerlikowske K. (2023). Loss of PPARγ activity characterizes early protumorigenic stromal reprogramming and dictates the therapeutic window of opportunity. Proc. Natl. Acad. Sci. USA.

[11] Fang Y., Shen Z.Y., Zhan Y.Z., Feng X.C., Chen K.L., Li Y.S., Deng H.J., Pan S.M., Wu D.H., Ding Y. (2019). CD36 inhibits β-catenin/c-myc-mediated glycolysis through ubiquitination of GPC4 to repress colorectal tumorigenesis. Nat. Commun..

[12] Liao C.Y., Li G., Kang F.P., Lin C.F., Xie C.K., Wu Y.D., Hu J.F., Lin H.Y., Zhu S.C., Huang X.X. (2024). Necroptosis enhances ‘don’t eat me’ signal and induces macrophage extracellular traps to promote pancreatic cancer liver metastasis. Nat. Commun..

[13] Nishiga Y., Drainas A.P., Baron M., Bhattacharya D., Barkal A.A., Ahrari Y., Mancusi R., Ross J.B., Takahashi N., Thomas A. (2022). Radiotherapy in combination with CD47 blockade elicits a macrophage-mediated abscopal effect. Nat. Cancer.

[14] Hu L.Y., Zhuang W.T., Chen M.J., Liao J., Wu D.F., Zhang Y.X., Pang L.L., Huang Y.H., Mao T.Q., Yang M.J. (2024). EGFR Oncogenic Mutations in NSCLC Impair Macrophage Phagocytosis and Mediate Innate Immune Evasion Through Up-Regulation of CD47. J. Thorac. Oncol..

[15] Zhuang Z., Zhou J., Qiu M., Li J., Lin Z., Yi H., Liu X., Huang C., Tang B., Liu B. (2024). The Combination of Anti-CD47 Antibody with CTLA4 Blockade Enhances Anti-Tumor Immunity in Non-Small Cell Lung Cancer via Normalization of Tumor Vasculature and Reprogramming of the Immune Microenvironment. Cancers.

[16] Sun Q., Zhang W., Wang L., Guo F., Song D., Zhang Q., Zhang D., Fan Y., Wang J. (2018). Hypermethylated CD36 gene affected the progression of lung cancer. Gene.

[17] Huang Q., Fan L., Gong M., Ren J., Chen C., Xie S. (2024). Metabolic reprogramming in lung cancer and its clinical implication. Front. Pharmacol..

[18] Liu L.Z., Wang B., Zhang R., Wu Z., Huang Y., Zhang X., Zhou J., Yi J., Shen J., Li M.Y. (2023). The activated CD36-Src axis promotes lung adenocarcinoma cell proliferation and actin remodeling-involved metastasis in high-fat environment. Cell Death Dis..

[19] Hall A. (1998). Rho GTPases and the actin cytoskeleton. Science.

[20] Ni K., Wang D., Xu H., Mei F., Wu C., Liu Z., Zhou B. (2019). miR-21 promotes non-small cell lung cancer cells growth by regulating fatty acid metabolism. Cancer Cell Int..

[21] Babaei G., Aziz S.G., Jaghi N.Z.Z. (2021). EMT, cancer stem cells and autophagy; The three main axes of metastasis. Biomed. Pharmacother..

[22] Shi Y., Zhao D., Xiao Z., Wang Y., Feng Q., Gu Y. (2025). The impact of miRNAs on epithelial-mesenchymal transition in lung cancer and the latest advances in their use as diagnostic markers. J. Cancer Res. Clin. Oncol..

[23] Young S.G., Song W., Yang Y., Birrane G., Jiang H., Beigneux A.P., Ploug M., Fong L.G. (2022). A protein of capillary endothelial cells, GPIHBP1, is crucial for plasma triglyceride metabolism. Proc. Natl. Acad. Sci. USA.

[24] Liu W., Qiao D., Chen J., Gao Y., Okuda K., Shimada Y., Yao L. (2025). Aspirin impedes non-small cell lung cancer development via fine-tuning the CD36 localization regulated by GPIHBP1. Transl. Lung Cancer Res..

[25] Chen J., Alduais Y., Zhang K., Zhu X., Chen B. (2021). CCAT1/FABP5 promotes tumour progression through mediating fatty acid metabolism and stabilizing PI3K/AKT/mTOR signalling in lung adenocarcinoma. J. Cell. Mol. Med..

[26] Bejarano L., Jordāo M.J.C., Joyce J.A. (2021). Therapeutic Targeting of the Tumor Microenvironment. Cancer Discov..

[27] Liao X., Yan S., Li J., Jiang C., Huang S., Liu S., Zou X., Zhang G., Zou J., Liu Q. (2022). CD36 and Its Role in Regulating the Tumor Microenvironment. Curr. Oncol..

[28] Shapouri-Moghaddam A., Mohammadian S., Vazini H., Taghadosi M., Esmaeili S.A., Mardani F., Seifi B., Mohammadi A., Afshari J.T., Sahebkar A. (2018). Macrophage plasticity, polarization, and function in health and disease. J. Cell. Physiol..

[29] Xu Z., Kuhlmann-Hogan A., Xu S., Tseng H., Chen D., Tan S., Sun M., Tripple V., Bosenberg M., Miller-Jensen K. (2025). Scavenger Receptor CD36 in Tumor-Associated Macrophages Promotes Cancer Progression by Dampening Type-I IFN Signaling. Cancer Res..

[30] Kadomoto S., Izumi K., Mizokami A. (2021). Macrophage Polarity and Disease Control. Int. J. Mol. Sci..

[31] Yin X., Sun X., Li A., Ruan J., Niu H., Zhou Y., Chen G., Guo J., He Q., Ji X. (2025). Wenxia Changfu formula inhibits Lewis lung cancer metastasis by reprogramming tumor-associated macrophages through the PPAR-γ/CD36 pathway. J. Ethnopharmacol..

[32] Shi J.H., Liu L.N., Song D.D., Liu W.W., Ling C., Wu F.X., Wang T.T., Liu B., Cui N.P., Qin Y. (2023). TRAF3/STAT6 axis regulates macrophage polarization and tumor progression. Cell Death Differ..

[33] Shi H., Chen S., Chi H. (2024). Immunometabolism of CD8(+) T cell differentiation in cancer. Trends Cancer.

[34] Xu S., Chaudhary O., Rodríguez-Morales P., Sun X., Chen D., Zappasodi R., Xu Z., Pinto A.F.M., Williams A., Schulze I. (2021). Uptake of oxidized lipids by the scavenger receptor CD36 promotes lipid peroxidation and dysfunction in CD8(+) T cells in tumors. Immunity.

[35] Guo D., Cai S., Deng L., Xu W., Fu S., Lin Y., Jiang T., Li Q., Shen Z., Zhang J. (2025). Ferroptosis in Pulmonary Disease and Lung Cancer: Molecular Mechanisms, Crosstalk Regulation, and Therapeutic Strategies. MedComm (2020).

[36] Qin Y., Huo F., Feng Z., Hou J., Ding Y., Wang Q., Gui Y., Yang Z., Yang J., Zhou G. (2025). CD36 promotes iron accumulation and dysfunction in CD8+ T cells via the p38-CEBPB-TfR1 axis in early-stage hepatocellular carcinoma. Clin. Mol. Hepatol..

[37] Lin Z., Zou S., Wen K. (2023). The crosstalk of CD8+ T cells and ferroptosis in cancer. Front. Immunol..

[38] Ma X., Xiao L., Liu L., Ye L., Su P., Bi E., Wang Q., Yang M., Qian J., Yi Q. (2021). CD36-mediated ferroptosis dampens intratumoral CD8(+) T cell effector function and impairs their antitumor ability. Cell Metab..

[39] Ma X., Sun Z., Chen H., Cao L., Zhao S., Fan L., Zhao C., Yin S., Hu H. (2024). 18β-glycyrrhetinic acid suppresses Lewis lung cancer growth through protecting immune cells from ferroptosis. Cancer Chemother. Pharmacol..

[40] Ao Y.Q., Gao J., Zhang L.X., Deng J., Wang S., Lin M., Wang H.K., Ding J.Y., Jiang J.H. (2023). Tumor-infiltrating CD36(+)CD8(+)T cells determine exhausted tumor microenvironment and correlate with inferior response to chemotherapy in non-small cell lung cancer. BMC Cancer.

[41] Subramanian M., Marelli-Berg F.M. (2021). CD36 pumps fat to defang killer T cells in tumors. Cell Metab..

[42] Chen P., Wang H., Tang Z., Shi J., Cheng L., Zhao C., Li X., Zhou C. (2025). Selective Depletion of CCR8+Treg Cells Enhances the Antitumor Immunity of Cytotoxic T Cells in Lung Cancer by Dendritic Cells. J. Thorac. Oncol..

[43] Wang H., Franco F., Tsui Y.C., Xie X., Trefny M.P., Zappasodi R., Mohmood S.R., Fernández-García J., Tsai C.H., Schulze I. (2020). CD36-mediated metabolic adaptation supports regulatory T cell survival and function in tumors. Nat. Immunol..

[44] Cabezón-Gutiérrez L., Palka-Kotlowska M., Custodio-Cabello S., Chacón-Ovejero B., Pacheco-Barcia V. (2025). Metabolic mechanisms of immunotherapy resistance. Explor. Target. Antitumor Ther..

[45] Singh S., Singh A.P., Mitra R. (2024). Cancer-Associated Fibroblasts: Major Co-Conspirators in Tumor Development. Cancers.

[46] Shintani Y., Kimura T., Funaki S., Ose N., Kanou T., Fukui E. (2023). Therapeutic Targeting of Cancer-Associated Fibroblasts in the Non-Small Cell Lung Cancer Tumor Microenvironment. Cancers.

[47] Li D., Yu H., Guo Z., Li S., Li Y., Guo Y., Zhong H., Xiong H., Liu Z. (2020). SERS analysis of carcinoma-associated fibroblasts in a tumor microenvironment based on targeted 2D nanosheets. Nanoscale.

[48] Kim B.G., Park K., Hwang M., Lee H., Park K.M., Choe J., Shin S.H., Jeong B.H., Lee K., Lee J. (2025). Identification of GREM-1 and GAS6 as Specific Biomarkers for Cancer-Associated Fibroblasts Derived from Patients with Non-Small-Cell Lung Cancer. Cancers.

[49] Chu L.Y., Ramakrishnan D.P., Silverstein R.L. (2013). Thrombospondin-1 modulates VEGF signaling via CD36 by recruiting SHP-1 to VEGFR2 complex in microvascular endothelial cells. Blood.

[50] Mehan M.R., Ayers D., Thirstrup D., Xiong W., Ostroff R.M., Brody E.N., Walker J.J., Gold L., Jarvis T.C., Janjic N. (2012). Protein signature of lung cancer tissues. PLoS ONE.

[51] Soto-Pantoja D.R., Kaur S., Roberts D.D. (2015). CD47 signaling pathways controlling cellular differentiation and responses to stress. Crit. Rev. Biochem. Mol. Biol..

[52] Lawler P.R., Lawler J. (2012). Molecular basis for the regulation of angiogenesis by thrombospondin-1 and -2. Cold Spring Harb. Perspect. Med..

[53] Isenberg J.S., Jia Y., Fukuyama J., Switzer C.H., Wink D.A., Roberts D.D. (2007). Thrombospondin-1 inhibits nitric oxide signaling via CD36 by inhibiting myristic acid uptake. J. Biol. Chem..

[54] Reiher F.K., Volpert O.V., Jimenez B., Crawford S.E., Dinney C.P., Henkin J., Haviv F., Bouck N.P., Campbell S.C. (2002). Inhibition of tumor growth by systemic treatment with thrombospondin-1 peptide mimetics. Int. J. Cancer.

[55] Huang J., Wang C., Hou Y., Tian Y., Li Y., Zhang H., Zhang L., Li W. (2023). Molecular mechanisms of Thrombospondin-2 modulates tumor vasculogenic mimicry by PI3K/AKT/mTOR signaling pathway. Biomed. Pharmacother..

[56] Martini C., DeNichilo M., King D.P., Cockshell M.P., Ebert B., Dale B., Ebert L.M., Woods A., Bonder C.S. (2021). CD36 promotes vasculogenic mimicry in melanoma by mediating adhesion to the extracellular matrix. BMC Cancer.

[57] Hale J.S., Li M., Sinyuk M., Jahnen-Dechent W., Lathia J.D., Silverstein R.L. (2012). Context dependent role of the CD36-thrombospondin-histidine-rich glycoprotein axis in tumor angiogenesis and growth. PLoS ONE.

[58] Lin Q., Choyke P.L., Sato N. (2023). Visualizing vasculature and its response to therapy in the tumor microenvironment. Theranostics.

[59] Qian C., Zhou Y., Zhang T., Dong G., Song M., Tang Y., Wei Z., Yu S., Shen Q., Chen W. (2024). Targeting PKM2 signaling cascade with salvianic acid A normalizes tumor blood vessels to facilitate chemotherapeutic drug delivery. Acta Pharm. Sin. B.

[60] Kane K., Edwards D., Chen J. (2025). The influence of endothelial metabolic reprogramming on the tumor microenvironment. Oncogene.

[61] Tan Y., Li J., Zhao G., Huang K.C., Cardenas H., Wang Y., Matei D., Cheng J.X. (2022). Metabolic reprogramming from glycolysis to fatty acid uptake and beta-oxidation in platinum-resistant cancer cells. Nat. Commun..

[62] Liu Y.Y., Huang W.L., Wang S.T., Hsu H.P., Kao T.C., Chung W.P., Young K.C. (2025). CD36 inhibition enhances the anti-proliferative effects of PI3K inhibitors in PTEN-loss anti-HER2 resistant breast cancer cells. Cancer Metab..

[63] Hajri T., Zaiou M., Fungwe T.V., Ouguerram K., Besong S. (2021). Epigenetic Regulation of Peroxisome Proliferator-Activated Receptor Gamma Mediates High-Fat Diet-Induced Non-Alcoholic Fatty Liver Disease. Cells.

[64] Huang H., Campbell S.C., Bedford D.F., Nelius T., Veliceasa D., Shroff E.H., Henkin J., Schneider A., Bouck N., Volpert O.V. (2004). Peroxisome proliferator-activated receptor gamma ligands improve the antitumor efficacy of thrombospondin peptide ABT510. Mol. Cancer Res..

[65] McCarty M.F., Barroso-Aranda J., Contreras F. (2008). PPAR gamma agonists can be expected to potentiate the efficacy of metronomic chemotherapy through CD36 up-regulation. Med. Hypotheses.

[66] Keshamouni V.G., Arenberg D.A., Reddy R.C., Newstead M.J., Anthwal S., Standiford T.J. (2005). PPAR-gamma activation inhibits angiogenesis by blocking ELR+CXC chemokine production in non-small cell lung cancer. Neoplasia.

[67] Jin N., Qian Y.Y., Jiao X.F., Wang Z., Li X., Pan W., Jiang J.K., Huang P., Wang S.Y., Jin P. (2025). Niraparib restricts intraperitoneal metastases of ovarian cancer by eliciting CD36-dependent ferroptosis. Redox Biol..

[68] Yang B., Tang H., Wang N., Gu J., Wang Q. (2023). Targeted DNA demethylation of the ZNF334 promoter inhibits colorectal cancer growth. Cell Death Dis..

[69] Su P.L., Furuya N., Asrar A., Rolfo C., Li Z., Carbone D.P., He K. (2025). Recent advances in therapeutic strategies for non-small cell lung cancer. J. Hematol. Oncol..

[70] Zhou X., Su M., Lu J., Li D., Niu X., Wang Y. (2024). CD36: The Bridge between Lipids and Tumors. Molecules.

[71] Wang Z., Pan B.S., Manne R.K., Chen J., Lv D., Wang M., Tran P., Weldemichael T., Yan W., Zhou H. (2025). CD36-mediated endocytosis of proteolysis-targeting chimeras. Cell.

[72] Sang C.Y., Liu J.R., Zheng Y.D., Chai T., Shi J.T., Naghavi M.R., Alibekovna K.E., Solievich B.A., Yang J.L. (2025). Obacunone potentiated PD-1 immunotherapy in pancreatic cancer by mediating CD36. Eur. J. Pharmacol..

[73] Zeng A., Chen H., Luo T., Chen W., Song Y., Xu Y., Chen Z., Tang Q., Zhu X., Deng C. (2025). Targeting OxLDL-mediated CD36 + CAF reprogramming potentiates PD-1 immunotherapy in osteosarcoma. Mol. Cancer.

[74] Jiang Q., Chen Z., Meng F., Zhang H., Chen H., Xue J., Shen X., Liu T., Dong L., Zhang S. (2023). CD36-BATF2\MYB Axis Predicts Anti-PD-1 Immunotherapy Response in Gastric Cancer. Int. J. Biol. Sci..

